# Treatment approaches for FGFR-altered urothelial carcinoma: targeted therapies and immunotherapy

**DOI:** 10.3389/fimmu.2023.1258388

**Published:** 2023-08-22

**Authors:** David J. Benjamin, Robert Hsu

**Affiliations:** ^1^ Hoag Family Cancer Institute, Newport Beach, CA, United States; ^2^ Department of Internal Medicine, Division of Medical Oncology, Norris Comprehensive Cancer Center, University of Southern California, Los Angeles, CA, United States

**Keywords:** FGFR, urothelial carcinoma, targeted therapy, erdafitinib, acquired resistance

## Abstract

The treatment of metastatic urothelial carcinoma has dramatically changed over the past decade with the approval of several therapies from multiple drug classes including immune checkpoint inhibitors, targeted therapies, and antibody drug conjugates. Although next generation sequencing of urothelial carcinoma has revealed multiple recurring mutations, only one targeted therapy has been developed and approved to date. Erdafitinib, a pan-fibroblast growth factor receptor (FGFR) inhibitor, has been approved for treating patients with select FGFR2 and FGFR3 alterations and fusions since 2019. Since then, emerging data has demonstrated efficacy of combining erdafitinib with immunotherapy in treating FGFR-altered urothelial carcinoma. Ongoing trials are evaluating the use of erdafitinib in non-muscle invasive urothelial carcinoma as well as in combination with enfortumab vedotin in the metastatic setting, while other FGFR targeted agents such as infigratinib, AZD4547, rogaratinib and pemigatinib continue to be in development. Future challenges will include strategies to overcome FGFR acquired resistance and efficacy and safety of combination therapies with erdafitinib and other FGFR targeted agents.

## Introduction

Urothelial carcinoma (UC), also referred to as bladder cancer, is a significant global public health concern with an estimated 573,000 cases and 213,000 deaths in 2020 alone (1). Tobacco use, occupational and environmental exposures to toxins such as aromatic amines, infections from schistosomiasis or recurrent urinary tract infections, and male gender are considered to be risk factors leading to the development of UC (2). UC is further classified as either muscle-invasive (MIBC) or non-muscle invasive (NMIBC), with the latter composing 75% of cases (3). Non-muscle invasive disease is generally managed with localized therapies including transurethral resection of bladder tumor (TURBT) followed by installation of intravesical Bacillus Calmette-Guerin (BCG) (4). BCG-refractory cases of NMIBC are evaluated for treatment with either nadofaragene firadenovec-vncg, pembrolizumab, or radical cystectomy (5, 6). Muscle-invasive UC management is dictated by a patient’s co-morbidities and ability to tolerate either chemotherapy and/or surgery with treatment modalities including: cystectomy with or without neoadjuvant chemotherapy and adjuvant nivolumab, tri-modality therapy with TURBT followed by chemotherapy plus radiation, or radiation therapy or TURBT alone (7). Despite treatment, approximately half of individuals with muscle-invasive disease may experience recurrence (8). Recurrence and metastatic UC is generally treated with systemic therapies including chemotherapy, immunotherapy, or antibody drug conjugates.

Despite multiple drug approvals for bladder cancer since 2016, the current standard of care in the first line setting for recurrent, locally advanced, or metastatic UC remains platinum-based chemotherapy with regimens including MVAC (methotrexate, vinblastine, doxorubicin, and cisplatin) and GC (cisplatin plus gemcitabine) (9, 10). Individuals who are not eligible for platinum-based chemotherapy can be treated with enfortumab vedotin, an antibody drug conjugate targeting nectin-4, plus pembrolizumab or with pembrolizumab alone (11, 12). Multiple somatic mutations have frequently been identified in UC including RB1, TP53, and TERT; however, only select FGFR2/3 alterations and fusions have a corresponding targeted therapy: erdafitinib (13, 14). In this review, we discuss the role of the FGFR pathway in the development of UC, in addition to evaluating established and developing treatment strategies in FGFR-altered UC.

## The FGFR pathway in urothelial carcinoma

UC has historically been categorized by histology including transitional cell, squamous cell, plasmacytoid, and sarcomatoid among other more rare variants (15). However, with the advent of gene expression analysis, both The Cancer Genome Atlas (TCGA) and Bladder Cancer Taxonomy Group have classified UC into several subtypes. According to TCGA analysis, UC is classified into four subtypes including luminal cluster I (30-35%), luminal cluster II (30-35%), basal cluster III (20-25%), and basal cluster IV (10-15%) (16). Moreover, luminal cluster I was found to commonly harbor FGFR3 mutations. In addition, the Bladder Cancer Taxonomy Group identified six molecular classes of MIBC based off 1750 transcriptome profiles: basal/squamous (35%), luminal papillary (24%), luminal unstable (15%), stroma-rich (15%), luminal non-specified (8%), and neuroendocrine-like (3%) (17). Their analysis revealed that luminal papillary tumors commonly harbored FGFR3 translocations and mutations. Multiple studies have demonstrated that FGFR3 alterations are common in over half of early stage UC cases including pTa and pT1 disease (18–21). It is estimated that approximately 20% of cases of advanced/metastatic UC harbors mutations in FGFR3 with a corresponding potential therapeutic option through inhibition of the FGFR pathway (22).

The FGFR signaling pathway involves several transmembrane proteins with intracellular tyrosine kinase domains. These proteins are, in turn, derived from the expression of four genes, specifically FGFR1-4. FGFR3 specifically encodes protein that plays several critical physiological roles including in cell growth, angiogenesis and proliferation. Alterations and fusions of FGFR3 have been shown to activate downstream pathways such as the MAPK and PI3K/AKT pathways which play roles in cancer development and progression (23). Following the identification of the FGFR signaling pathway and its role in the development of UC when altered, there was significant interest in identifying molecules to inhibit this pathway. Ultimately, such research led to the identification of JNJ-42756493, now known as erdafitinib (24).

## Erdafitinib in platinum-refractory urothelial carcinoma

Erdafitinib is an oral pan-FGFR (FGFR1-4) tyrosine kinase inhibitor (TKI) which causes prolonged inhibition of the FGFR pathway signaling owing to uptake in intra-cellular lysosomes (24). Pre-clinical data demonstrated anti-tumor activity in both xenograft models from human tumor cells as well as tumor tissue from individuals with cancer. Based off this promising pre-clinical activity as well as a phase I study evaluating the FGFR inhibitor in individuals with advanced or refractory solid tumors harboring FGFR mutations, erdafitinib was studied in 99 patients with locally advanced and unresectable or metastatic UC in the BLC2001 study (25, 26). Eligible patients in this open-label, phase II, single arm study had either qualifying translocations (FGFR2-BICC1, FGFR2-CASP7, FGFR3-TACC3, FGFR3-BAIAP2L1) or FGFR3 gene mutations (R248C, S249C, G370C, Y373C). In addition, individuals were required to have disease progression after at least prior course of systemic chemotherapy or within 12 months after receiving either neoadjuvant or adjuvant chemotherapy. Of note, patients were allowed to have previously received immunotherapy. Based off interim safety and efficacy analysis, the trial protocol was amended to a starting dose of 8 mg per day with escalation to 9 mg daily dependent on no adverse effects at day 14 on treatment. BLC2001’s primary endpoint was response rate (RR), and secondary endpoints included progression-free survival (PFS), overall survival (OS), duration of response, and RR in biomarker-specific subgroups. 

At the cutoff date for primary analysis on March 15, 2018, BLC2001 reported the response rate was 40% (95% confidence interval [CI], 31-50) with 3% of individuals having a complete response and 37% having a partial response. An additional 39% of patients had stable disease, while 18% of patients experienced progressive disease. The median time to response was 1.4 months. Subgroup analysis demonstrated among individuals with FGFR3 mutations, 36 of 74 patients had a treatment response while 4 of 25 patients with FGFR2/3 fusions had a response. With regards to the trial’s secondary endpoints, the median duration of response was 5.6 months (95% CI, 4.2-7.2). In addition, median PFS was 5.5 months (95% CI, 4.2-6.0) and median OS was 13.8 months (95% CI, 9.8 to not reached).

Approximately two-thirds (67%) of patients enrolled in BLC2001 experienced grade 3 or 4 adverse events with 46% of grade 3 or higher events considered to be treatment-related. The most common adverse events of any grade include: hyperphosphatemia (77%), stomatitis (58%), diarrhea (51%), dry mouth (46%), decreased appetite (38%), dysgeusia (37%), fatigue (32%), dry skin (32%) and alopecia (29%). Ocular toxicities of any grade occurred in 51% of patients including: central serous retinopathy (21%), dry eye (19%), blurred vision (17%), cataracts (6%), and keratitis (5%). The most common grade 3 or higher adverse events include: hyponatremia (11%), stomatitis (10%), asthenia (7%), nail dystrophy (5%), and hand foot syndrome (5%). Although hyperphosphatemia has been considered a side effect due to “on-target” effects from FGFR inhibition, data from FGFR inhibitor infigratinib is suggestive that hyperphosphatemia may be a surrogate biomarker for treatment response, with improved response rates among individuals who experienced hyperphosphatemia (27).

Given BLC2001 met its primary end point in response rate and due to its safety profile, the US Food and Drug Administration (FDA) granted accelerated approval to erdafitinib on April 12, 2019 for use in individuals with metastatic UC who progressed during or following platinum-based chemotherapy. Given the promising result in the phase II BLC2001 trial, additional studies comparing erdafitinib to chemotherapy and erdafitinib in combination with immunotherapy were undertaken with the THOR trial and NORSE trial, respectively.

## Erdafitinib versus chemotherapy in urothelial carcinoma

The phase III randomized THOR trial compared erdafitinib to investigator’s choice of chemotherapy (either docetaxel or vinflunine) in UC with FGFR2/3 alterations that was previously treated with one or two prior lines of systemic therapy. The study’s primary end point was OS and secondary endpoints included PFS, objective response rate (ORR) and safety profile. Among 266 enrolled patients, 136 were assigned to receive erdafitinib while 130 patients received chemotherapy (28). The trial results, which were presented at the 2023 ASCO Annual Meeting demonstrated that erdafitinib significantly improved OS with a median OS of 12.1 months among those receiving erdafitinib compared to 7.8 months in those receiving chemotherapy (HR 0.68, 95% 0.47-0.88, p = 0.0050). In addition, among secondary endpoints, median PFS was significantly improved with erdafitinib at 5.6 months compared to 2.7 months with chemotherapy (HR 0.58, 95% CI 0.44-0.78, p = 0.0002). In addition, individuals who received erdafitinib were more likely to respond to treatment with an ORR of 46% in comparison to 12% with chemotherapy (relative risk 3.94, 95% CI 2.37-5.67, p < 0.001).

The THOR trial demonstrated similar rates of grade 3 or higher adverse events with an occurrence of 46% in each trial arms, with 1 death with erdafitinib and 6 deaths with chemotherapy. Of note, central serous retinopathy occurred 23% of patients who received erdafitinib in concordance to a similar rate of incidence found with erdafitinib as studied in the BLC2001 study. Given its superiority to chemotherapy as well as its safety profile demonstrated in the phase III THOR trial, erdafitinib is anticipated to receive full approval for treatment of platinum-refractory metastatic UC.

## The role of erdafitinib plus immunotherapy

Luminal 1 subtype UC tumors, which harbor FGFR3 mutations, have been shown to lack immune cell infiltration and immune marker expression (29). As such, in comparison to other UC subtypes, luminal 1 subtype tumors have shown to have the lowest response rate to immune checkpoint inhibitors such as atezolizumab and nivolumab (30, 31). However, pre-clinical data involving a genetically engineered murine model with lung cancer harboring FGFR mutations showed that treatment with erdafitinib led to CD4+ and CD8+ T cell infiltration of the tumor (32). In addition, it was shown that erdafitinib monotherapy led to a decrease in the number of Tregs in tumors. As such, this study revealed that treating FGFR altered tumors may alter the tumor microenvironment from one that is “cold” to one that is “hot” and therefore may benefit from immune checkpoint inhibitors when combined with erdafitinib.

The phase II NORSE trial evaluated the efficacy of erdafitinib in combination with immune checkpoint inhibitor cetrelimab in treatment-naïve individuals with metastatic UC who were ineligible for cisplatin-based chemotherapy. Patients were randomized to receive either erdafitinib or erdafitinib in combination with cetrelimab (given at 240 mg every 2 weeks for the first four cycles followed by 480 every 4 weeks). The study, which was presented at the 2023 ASCO Annual Meeting, designated ORR as the primary endpoint with duration of response, PFS, OS and time to response as secondary endpoints. Among 87 patients enrolled, 43 received erdafitinib monotherapy while 44 patients received erdafitinib plus immunotherapy combination therapy. The ORR for monotherapy was 44.2% with one patient experienced a complete response, whereas the ORR for combination therapy was 54.5% with six (13.6%) complete responses (33). Among secondary endpoints, median PFS was longer with combination therapy at 10.97 months (95% CI, 5.45-13.63) in comparison to 5.62 months (95% CI, 4.34-7.36) with erdafitinib monotherapy. The most common treatment-related adverse events included hyperphosphatemia (68.9% versus 83.7%), stomatitis (59.1% versus 72.1%) and diarrhea (45.5% versus 48.8%) in the combination therapy and monotherapy arms, respectively. Of note, one patient death was reported in the combination arm due to respiratory failure related to cetrelimab therapy. Although cetrelimab currently has not received FDA approval for any oncologic condition, the NORSE trial demonstrated efficacy with combination erdafitinib plus immune checkpoint inhibitor treatment in a patient population with otherwise limited treatment options. **Table 1** shows all completed prospective clinical trials evaluating erdafitinib in FGFR-altered urothelial carcinoma.

**Table 1:** Completed trials evaluating erdafitinib in metastatic urothelial carcinoma.

Trial Name	Therapy/Therapies	Primary Endpoint	Secondary Endpoints	Safety Profile	Reference
BLC2001	Erdafitinib	Response rate was 40% (95% confidence interval [CI], 31-50) with 3% complete response and 37% partial response	Median PFS was 5.5 months (95% CI, 4.2-6.0) and median OS was 13.8 months (95% CI, 9.8 to not reached).	Most common adverse events of any grade include: hyperphosphatemia (77%), stomatitis (58%), diarrhea (51%), dry mouth (46%), decreased appetite (38%), dysgeusia (37%), and fatigue (32%). Ocular toxicities of any grade occurred in 51% of patients including: central serous retinopathy (21%), dry eye (19%), and blurred vision (17%).	(26)
THOR	Erdafitinib versus chemotherapy (docetaxel or vinflunine)	Median OS was 12.1 months with erdafitinib and 7.8 months with chemotherapy (HR 0.68, 95% 0.47-0.88, p = 0.0050)	Median PFS was 5.6 months with erdafitinib at 5.6 months and 2.7 months with chemotherapy (HR 0.58, 95% CI 0.44-0.78, p = 0.0002). ORR of 46% with erdafitinib and 12% with chemotherapy (relative risk 3.94, 95% CI 2.37-5.67, p < 0.001)	Grade 3 or higher adverse events with an occurrence of 46% in each trial arms, with 1 death with erdafitinib and 6 deaths with chemotherapy. Central serous retinopathy occurred in 23% of patients who received erdafitinib	(28)
NORSE	Erdafitinib plus cetrelimab	ORR for combination therapy was 54.5% with six (13.6%) complete responses; ORR for monotherapy was 44.2% with one complete response	Median PFS with combination therapy was 10.97 months (95% CI, 5.45-13.63) and 5.62 months (95% CI, 4.34-7.36) with erdafitinib monotherapy	Treatment-related adverse events included hyperphosphatemia (68.9% versus 83.7%), stomatitis (59.1% versus 72.1%) and diarrhea (45.5% versus 48.8%) in the combination therapy and monotherapy arms, respectively	(33)

## Other FGFR-directed therapies

Other agents targeting FGFR signaling include infigratinib and AZD4547, which are oral FGFR1-3 inhibitors and rogaratinib and pemigatinib, which are oral FGFR1-4 selective inhibitors. Infigratinib showed an ORR of 25.4% in 67 patients with metastatic platinum-refractory, FGFR-mutated UC (34). Of note, among 8 patients with upper tract UC, 1 patient had a complete response and 3 had partial responses on follow-up radiographic imaging. Currently, there is an ongoing, randomized, placebo-controlled phase 3 PROOF-302 trial evaluating the use of adjuvant infigratinib in invasive UC in FGFR3 mutated patients who are ineligible for/or refusing cisplatin chemotherapy (ClinicalTrials.gov identifier NCT04197986) (35).

AZD4547 demonstrated activity in a phase 1I study involving tumors with aberrations of the FGFR pathway with partial responses seen in 8% of 48 patients on therapy (36). Of note, in the study, the second most common primary tumor was urothelial carcinoma (12.5%). There is currently an ongoing phase II trial looking at safety and efficacy of AZD4547 with tislelizumab, a humanized IgG4 anti-PD-1 inhibitor in advanced UC (ClinicalTrials.gov identifier NCT05775874).

The FORT-1 trial was a phase II/III study comparing rogaratinib to chemotherapy (docetaxel, paclitaxel or vinflunine) in metastatic FGFR1/3 altered UC (37). The overall response rate for treatment with the FGFR inhibitor (20.7%) was similar to chemotherapy (19.3%). However, the trial failed to demonstrate improved survival with rogaratinib as the median OS with the FGFR inhibitor was 8.3 months versus 9.8 months with chemotherapy (hazard ratio 1.11; 95% CI, 0.71-1.72, p =0.67). Given the negative data with survival, it is unlikely that rogaratinib will receive approval.

Pemigatinib is currently FDA approved for metastatic cholangiocarcinoma with FGFR2 alterations and in relapsed/refractory myeloid/lymphoid neoplasms with FGFR1 rearrangement (38). The FGFR 1-3 inhibitor was studied in 128 patients with refractory advanced malignancies harboring FGF/FGFR gene alterations. In the study, 12 partial responses were seen including in one patient with advanced urothelial carcinoma (39). FIGHT-205 investigated pemigatinib with pembrolizumab versus pemigatinib alone versus standard of care in metastatic FGFR3 mutant UC who are cisplatin-ineligible, but the study was discontinued to business reasons (ClinicalTrials.gov identifier NCT04003610).

## Future directions in treating FGFR-altered UC

Given the efficacy of erdafitinib in treating FGFR-altered UC, several ongoing trials are evaluating the use of erdafitinib in a multitude of treatment settings. Erdafitinib is currently being studied in a phase II trial comparing 2 years of erdafitinib with investigator’s choice of intravesical chemotherapy (gemcitabine or mitomycin C) in individuals with previously BCG-treated, recurrent high risk NMIBC (ClinicalTrials.gov identifier NCT04172675). In addition, erdafitinib is being studied through an intravesical delivery system known as TAR-210 in individuals with selected FGFR mutations and fusions. In the dose escalation phase, eligible participants will include individuals with previously BCG-treated, recurrent high-risk papillary NMIBC who either refuse or are ineligible for cystectomy. As part of the part 2 dose expansion, participants will be placed into NMIBC and MIBC cohorts (ClinicalTrials.gov identifier NCT05316155) (40). One potential challenge may be the duration of treatment given the systemic side effects associated with erdafitinib therapy.

Erdafitinib is further being evaluated in the treatment of MIBC as well. The ongoing NERA trial is evaluating the use of erdafitinib in individuals with MIBC harboring select FGFR2/3 alterations who are ineligible for cisplatin-based chemotherapy. In the trial, patients will receive neoadjuvant erdafitinib with or without atezolizumab. The NERA trial’s primary endpoint will be evaluating for pathologic complete response (pCR) rates at the time of cystectomy (ClinicalTrials.gov identifier NCT05564416). Finally, erdafitinib is being studied in combination with enfortumab vedotin (EV) in a phase Ib trial for patients with FGFR2/3-altered mUC refractory to chemotherapy and immunotherapy. The study’s primary objectives include determining the feasibility and safety of combination erdafitinib and EV, as well as determining the maximum tolerated dose (MTD) as well as recommended phase 2 dose (RP2D) (ClinicalTrials.gov identifier NCT04963153). **Table 2** shows all ongoing or planned clinical trials evaluating erdafitinib in FGFR-altered urothelial carcinoma.

**Table 2:** Ongoing or planned clinical trials evaluating erdafitinib in FGFR-Altered UC.

Disease Setting	Phase	Therapy/Therapies	Primary Endpoint(s)	ClinicalTrials.gov identifier
Previously BCG-treated, recurrent high risk NMIBC	II	Two years of oral erdafitinib versus intravesical chemotherapy (gemcitabine or mitomycin C)	Recurrence free survival (RFS)	NCT04172675
Previously BCG-treated, recurrent high-risk papillary NMIBC who either refuse or are ineligible for cystectomy (part 1); NMIBC and MIBC (part 2)	I	Intravesical erdafitinib delivery system (TAR-210)	Number of participants with adverse events and by severity	NCT05316155
Cisplatin-ineligible patients with MIBC	II	Erdafitinib with or without atezolizumab	Pathologic complete response (pCR) rates	NCT05564416
Metastatic UC refractory to chemotherapy and immunotherapy	Ib	Erdafitinib with enfortumab vedotin (EV)	Feasibility and safety of erdafitinib plus EV	NCT04963153

Finally, a commonly encountered clinical dilemma is acquired resistance to erdafitinib as well as other TKIs targeting the FGFR signaling pathway. Much of the acquired resistance to the FGFR pathway occurs from a compensatory signaling mechanism to FGFR inhibition. For example, the PI3K pathway has been identified as a pathway of resistance to FGFR inhibitor AZD4547 (41). The V561M mutation within the FGFR1 tyrosine kinase domain has been identified in the FGFR-driven leukemia patients and associated with acquired resistance to infigratinib and AZD4547 (42). Other resistance mechanisms include activation of pathways shared with FGFR such as EGFR, PDGFR, VEGFR, TRK, IGFR, Tie1,2 (43). Thus, new generation therapies addressing mutations such as GZD824 in overcoming FGFR1-V561M or consideration of a combination of EGFR and FGFR inhibitors in patients with EGFR T790M mutations may be potential avenues for additional combination therapies with FGFR inhibition moving forward (44, 45).

## Conclusion

FGFR alterations are commonly found in both NMIBC and MIBC. Erdafitinib has specifically demonstrated efficacy in previously treated mUC and is currently approved in treating mUC with specific FGFR2/3 alterations. Recent phase II also supports the use of erdafitinib in combination with immune checkpoint inhibition as another viable therapeutic avenue that warrants further investigation. Ongoing studies may allow for oral and/or intravesicular deliver of erdafitinib as a potential therapeutic option for recurrent NMIBC. Future therapies inhibiting the FGFR signaling pathway along with overcoming acquired patterns of FGFR resistance may provide continued improved outcomes for individuals with UC.

## Author contributions

DB: Conceptualization, Formal Analysis, Investigation, Methodology, Project administration, Resources, Visualization, Writing – original draft, Writing – review and editing. RH: Conceptualization, Formal Analysis, Funding acquisition, Investigation, Project administration, Resources, Writing – original draft, Writing – review and editing.
